# Kinetic Study on the Cs_*X*_H_*3−X*_ PW_12_O_40_/Fe-SiO_2_ Nanocatalyst for Biodiesel Production

**DOI:** 10.1155/2013/612712

**Published:** 2013-12-10

**Authors:** Mostafa Feyzi, Leila Norouzi, Hamid Reza Rafiee

**Affiliations:** ^1^Faculty of Chemistry, Razi University, P.O. Box 6714967346, Kermanshah, Iran; ^2^Nanoscience & Nanotechnology Research Center (NNRC), Razi University, P.O. Box 6714967346, Kermanshah, Iran

## Abstract

The kinetic of the transesterification reaction over the Cs_*X*_H_3*−X*_PW_12_O_40_/Fe-SiO_2_ catalyst prepared using sol-gel and impregnation procedures was investigated in different operational conditions. Experimental conditions were varied as follows: reaction temperature 323–333 K, methanol/oil molar ratio = 12/1, and the reaction time 0–240 min. The H_3_PW_12_O_40_ heteropolyacid has recently attracted significant attention due to its potential for application in the production of biodiesel, in either homogeneous or heterogeneous catalytic conditions. Although fatty acids esterification reaction has been known for some time, data is still scarce regarding kinetic and thermodynamic parameters, especially when catalyzed by nonconventional compounds such as H_3_PW_12_O_40_. Herein, a kinetic study utilizing Gc-Mas in situ allows for evaluating the effects of operation conditions on reaction rate and determining the activation energy along with thermodynamic constants including Δ*G*, Δ*S*, and Δ*H*. It indicated that the Cs_*X*_H_3*−X*_PW_12_O_40_/Fe-SiO_2_ magnetic nanocatalyst can be easily recycled with a little loss by magnetic field and can maintain higher catalytic activity and higher recovery even after being used 5 times. Characterization of catalyst was carried out by using scanning electron microscopy (SEM), X-ray diffraction (XRD), Fourier transform-infrared spectroscopy (FT-IR), N_2_ adsorption-desorption measurements methods, thermal gravimetric analysis (TGA), and differential scanning calorimetry (DSC).

## 1. Introduction

Biodiesel is a fuel composed of monoalkyl esters of long chain fatty acids derived from renewable sources, such as vegetable oils and animal fats [1]. Due to oxygen content, biodiesel is a clean, nontoxic, and biodegradable fuel with low exhaust emissions, without the sulphur and carcinogen content [2]. Biodiesel is prepared via reaction between triglycerides and alcohol in the presence of a catalyst [3]. Transesterification reaction of oil and alcohol with homogeneous catalyst is the most common method for the preparation of biodiesel [4, 5]. However, the homogeneous catalyst has many drawbacks, such as the difficulty in product separation, and equipment corrosion requirement of large quantity of water, environmental pollution [6]. The use of heterogeneous catalysts to replace homogeneous ones is easily regenerated and has a less corrosive nature, leading to safer, cheaper, and more environment-friendly operations, [7]. Currently, the research is focused on sustainable solid acid catalysts for transesterification reaction. In addition, it is believed that solid acid catalysts have the strong potential than liquid acid catalyst [8]. HPW catalyst is a strong Brønsted acid, with high thermal stability and high solubility in polar solvents, and it has shown to be more active in FFA esterification reactions than mineral acid catalysts [8–12]. However, heteropolyacids have low surface areas and high solubility in polar solvents. These features make some difficulties in catalyst recovery and catalyst lifetime [13]. Therefore, salts of HPAs with large single valence ions, such as Cs^+^, NH_4_^+^, and Ag^+^, have attracted much fondness because these salts will increase in surface area and profound changes in solubility over the parent heteropolyacid [14, 15]. HPW can be supported on several kinds of support such as SiO_2_, Al, ZrO_2_, activated carbon, SiO_2_-Al, and MCM-41 that SiO_2_ is cheap, easily available, and easily surface modifiable [16–18].

In the present kinetic study, pseudo-first-order model was applied to correlate the experimental kinetic data of catalytic performance of the Cs_*X*_H_3−*X*_PW_12_O_40_/Fe-SiO_2_ for sunflower oil transesterification with methanol and the main thermodynamic parameters such as the effects of temperature on reaction rate, activation energy, entropy variation (Δ*S*), and enthalpy variation (Δ*H*) were determined; moreover, the effects of temperature on reaction rate and the order of reaction were assessed. Characterization of catalyst was carried out by using scanning electron SEM, XRD, FT-IR, N_2_ adsorption-desorption measurements methods, TGA, and DSC methods.

## 2. Experimental

### 2.1. Fe-SiO_**2**_ Support Preparation

All materials with analytical purity were purchased from Merck and used without further purification. Ferric nitrate (Fe(NO_3_)_3_·9H_2_O) and tetraethyl orthosilicate (TEOS) were selected as the source of ferric and silica, respectively. A typical procedure for the preparation of ferric-silica mixed oxide containing 60 wt% ferric was followed. Firstly, 30 mL TEOS was mixed with certain amount anhydrous ethyl alcohol (C_2_H_5_OH). Secondly, 35.234 gr Fe(NO_3_)_3_·9H_2_O was dissolved with certain amount of anhydrous ethyl alcohol under stirring; also 30 gr of oxalic acid was dissolved in certain amount of anhydrous alcohol under stirring. In the final step, Fe and Si sols and oxalic acid were added simultaneously into the beaker under constant stirring to obtain a gel form. After the end of the above operations, the samples were aged for 90 min at 50°C. The obtained gel was dried in an oven (120°C, 12 h) to give a material denoted as the catalyst precursor. Finally, the catalyst precursor was calcined at 600°C for 6 h to produce magnetic solid catalyst. The Fe-SiO_2_ supported 12-tungstophosphoric acid catalyst with 4 wt.% of aqueous solution HPW (based on the Fe-SiO_2_ weight) were prepared by incipient wetness impregnation. The impregnated precursor was dried at 120°C for overnight and calcined at 600°C for 6 h.

Finally, the promoted catalyst by Cs was synthesized with Cs/H_3_PW_12_O_40_ = 2 wt.%. The salt obtained by this procedure will be designated hereinafter as Cs_*X*_H_3−*X*_PW_12_O_40_/Fe-SiO_2_, where *X* is the amount of protons per [PW_12_O_40_]^3−^ anion in the salt.

### 2.2. Characterization of Catalyst

#### 2.2.1. N_**2**_ Physisorption Measurements

The specific surface area, total pore volume, and the mean pore diameter were measured using a N_2_ adsorption-desorption isotherm at liquid nitrogen temperature (−196°C), using a NOVA 2200 instrument (Quantachrome, USA). Prior to the adsorption-desorption measurements, all the samples were degassed at 110°C in a N_2_ flow for 2 h to remove the moisture and other adsorbates.

#### 2.2.2. Scanning Electron Microscopy (SEM)

The morphology of catalyst and precursor was observed by means of an S-360 Oxford Eng scanning electron microscopy.

#### 2.2.3. Fourier Transform-Infrared Spectroscopy (FT-IR)

Fourier transform infrared (FT-IR) spectra of the samples were obtained using a Bruker Vector 22 spectrometer in the region of 400–4000 cm^−1^.

#### 2.2.4. X-Ray Diffraction (XRD)

X-ray diffraction (XRD) patterns of the catalysts were recorded on a diffractometer using CuK_*α*_ radiation. The intensity data were collected over a 2*θ* range of 15–75.

#### 2.2.5. Thermal Gravimetric Analysis (TGA) and Differential Scanning Calorimetry (DSC)

The TGA and DSC were carried out using simultaneous thermal analyzer (PerkinElmer) under a flow of dry air with a flow rate of 50 mL min^−1^. The temperature was raised from 20 to 700°C using a linear programmer at a heating rate of 3°C min^−1^.

### 2.3. Catalytic Tests

The type and quantity of methyl esters in the biodiesel samples were determined using gas chromatography-mass spectrometry (GC Agilent 6890N model and Mass Agilent 5973N model) equipped with a flame ionized detector (FID). A capillary column (HP-5) with column length (60 m), inner diameter (0.25 mm), and 0.25 *μ*m film thickness was used with helium as the carrier gas. The temperature program for the biodiesel samples started at 50°C and ramped to 150°C at 10°C min^−1^. The temperature was held at 150°C for 15 min and ramped to 280°C at 5°C min^−1^. The holding time at the final temperature (250°C) was 5 min. Also, the injector was used from kind split/splitless.

### 2.4. Kinetic Studies

The transesterification of sunflower oil was carried out in a round bottomed flask fitted with a condenser and magnetic stirring system. The reaction system was heated to selected temperature in 50, 55, and 60°C. When the oil reached selected temperature, methanol/oil molar ratio = 12/1 and the catalyst amount (3 wt% related to oil weight) were added with continuous stirring (500 rpm). After completion of the reaction time (0–240 min), the sample concentration is calculated according to mole fraction at any time. The results can be seen in Table 1.

## 3. Kinetic Model

The mole fraction for the transesterification reaction was established. For the reaction stoichiometry requires 3 Mol of methanol (M) and 1 mol of triglyceride (TG) to give 3 mol of methyl ester (ME) and 1 Mol of glycerol (GL). Transesterification reaction comprises three consecutive reversible reactions, where 1 mol of ME is produced in each step and monoglycerides (MG) and diglycerides (DG) are intermediate products [19]. The kinetic model used in this work is based on the following assumptions.

1. Firstly, *k*_eq_ should be considered not to depend on methanol concentration (due to its excess) (the reaction is considered pseudo-first order [20, 21]).
2. Production of intermediate species is negligible (the result reaction is a one step).
3. The chemical reaction occurred in the oil phase.

Based on assumption (1),

(1) $$\begin{array}{c} -r=\frac{-d\left[TG\right]\;}{dt}=k\left[TG\right]{\left[\text{ROH}\right]}^{3}. \end{array}$$

And based on assumption (2),

(2) k′=k[ROH]3−r=−d[TG]dt=k′[TG]⇒ln⁡TG0−ln⁡TG=k′·t.

According to the mass balance,

(3) $$\begin{array}{c} X_{\text{ME}}=1-\frac{\left[TG\right]}{\left[{TG}_{0}\right]}\\ \left[TG\right]=\left[TG_{0}\right]\left[1-X_{\text{ME}}\right]\\ \frac{{dX}_{\text{ME}}}{dt}=k^{\prime }\left[1-X_{\text{ME}}\right]\Rightarrow -\ln\left(1-X_{\text{ME}}\right)=k^{\prime }\cdot t. \end{array}$$

Based on this model and the experimental data, at first we calculated the concentration of methyl ester at different times (based on the moles fraction). Second, the rate constants at each temperature were obtained. Third, the preexponential factors and activation energies are obtained by plotting the logarithm of the rate constants (*k*) versus 1/*T* of absolute temperature using the Arrhenius equation and in the final stage thermodynamic parameters were obtained such as Δ*S* and Δ*H*.

## 4. Results and Discussion

### 4.1. Characterization of the Catalyst

**Figure 1:**
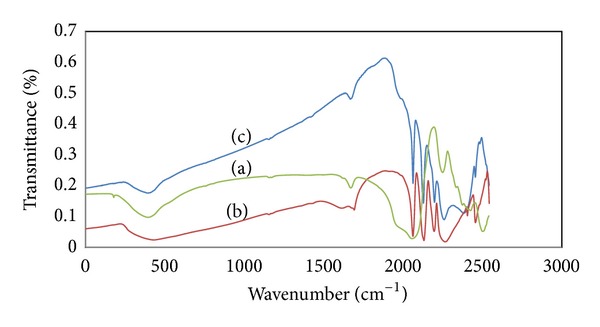
FT-IR spectrums of the Cs_*X*_H_3−*X*_PW_12_O_40_/Fe-SiO_2_ (a), H_3_PW_12_O_40_ (b), and Cs_*X*_H_3−*X*_PW_12_O_40_ (c) nanocatalysts.

The FT-IR spectra of H_3_PW_12_O_40_, Cs_*X*_PW_12_O_40_ and Cs_*X*_H_3−*X*_PW_12_O_40_/Fe-SiO_2_ are shown in Figure 1. The Keggin anion of HPW consists of a central phosphorous atom tetrahedrally coordinated by four oxygen atoms and surrounded by twelve octahedral WO_6_ units that share edges and corners in the structure.bands related to a Keggin structure (i(O–P–O) = 550 cm^−1^, indicative of the bending of the central oxygen of P–O–P; *ν*as(W–Oe–W) at 798 cm^−1^, related to asymmetric stretching of tungsten with edge oxygen in W–O–W; *ν*as(W–OcW) = 893 cm^−1^, related to the asymmetric stretching of corner oxygen in W–O–W; *ν*as(W=O) = 983 cm^−1^, indicative of the asymmetric stretching of the terminal oxygen; and *ν*(P–O) at 1080 cm^−1^, assigned to asymmetric stretching of oxygen with a central phosphorous atom) [22, 23]. HPA salts maintain their corner Keggin structure with the addition of different amount of metallic Cs. When Cs_*X*_H_3−*X*_PW_12_O_40_ is supported on Fe-SiO_2_, these bands have somewhat changed. The bands at 1080 and 890 cm^−1^ are overlapped by the characteristic band of SiO_2_, while these bands at 985 and 794 cm^−1^ shift to 966 and 805 cm^−1^, respectively.

**Figure 2:**
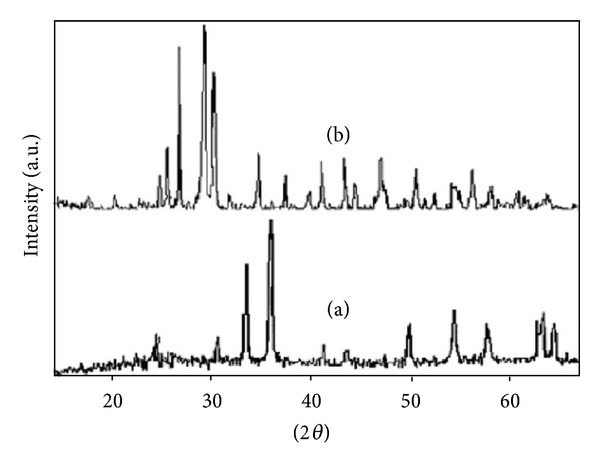
XRD patterns of the Cs_1_H_2_PW_12_O_40_/Fe-SiO_2_ (a) and Cs_1_H_2_PW_12_O_40_ (b) nanocatalysts.

The XRD pattern of Cs_*X*_H_3−*X*_PW_12_O_40_/Fe-SiO_2_ catalyst was presented in Figure 2. Supported HPA samples do not show diffraction patterns probably due to the following reasons: (i) after treatment at 500°C, the HPA practically loses its crystalline structure, (ii) HPA species were highly dispersed, and (iii) the deposited amount of HPA was not big enough to be detected by this technique [24].

**Figure 3:**
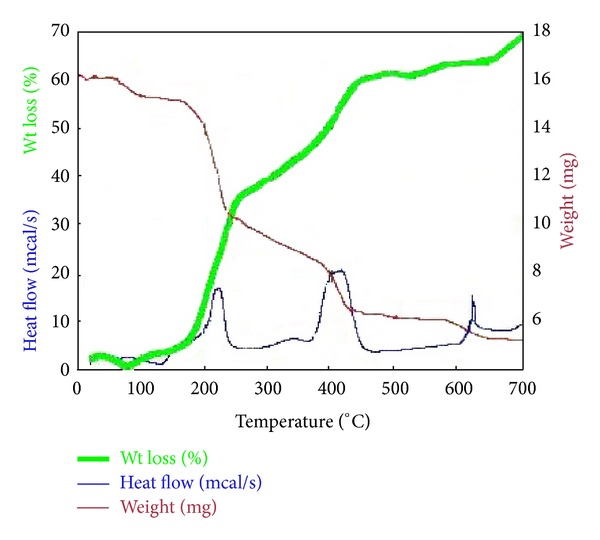
TGA and DSC curves for Cs_*X*_H_3−*X*_PW_12_O_40_/Fe-SiO_2_ precursor.

The TGA-DSC experiment on the catalyst precursor has shown four steps of mass loss (Figure 3). The first step at the temperature of 70–110°C was attributed to the evaporation of residual moistures in the catalyst precursor and loss of physisorbed waters. The second stage (190–280°C) is accompanying weight loss of the crystallization water expelling which is most likely the hydrated proton. The peak around 390–460°C is due to the decomposition of iron and Si oxalates to oxide phases. Most of weight loss happened from 580°C to 640°C due to the phase transition and formation of Fe_2_SiO_4_ (cubic). The weight loss curve is involved with a total overall weight loss of ca. 69 wt%. DSC measurement was performed in order to provide further evidence for the presence of the various species and evaluate their thermal behavior. As shown in Figure 3, the endothermic curve represents the removal of the physically adsorbed water from the material (70–110°C). Two exothermic peaks at around 190–280°C and 390–480°C are due to the crystallization water expelling which is most likely the hydrated proton and the decomposition of iron and Si oxalates to oxide phases, respectively. The exothermic peak at around 580–640°C is due to formation of iron silicate phase [25].

**Figure 4:**
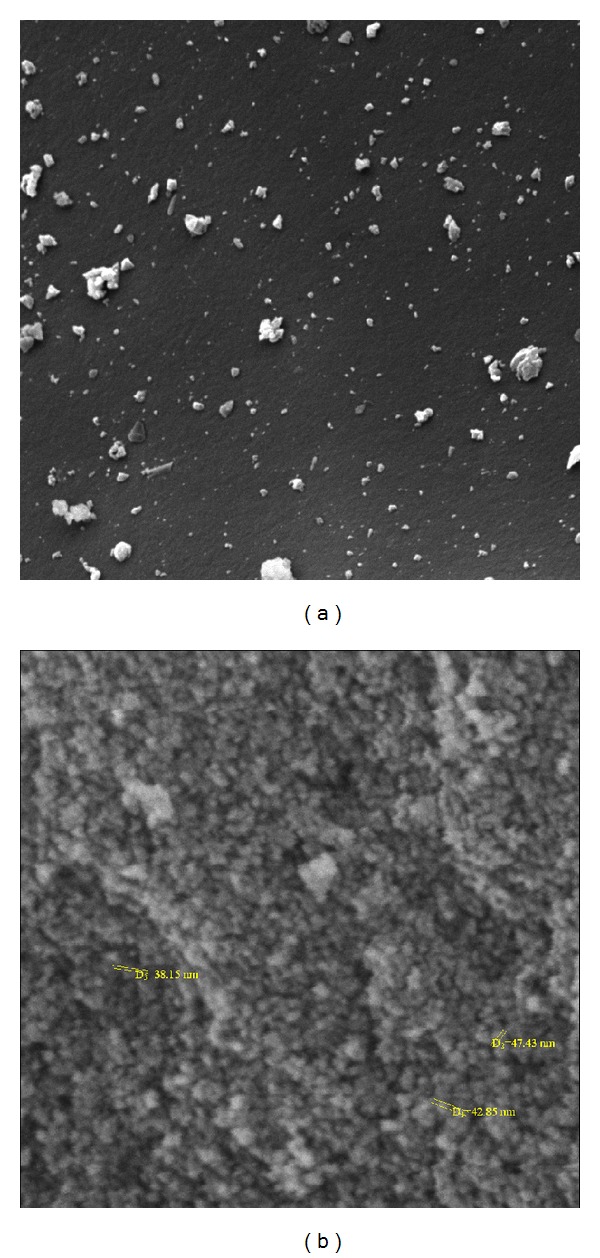
The SEM images of Cs_*X*_H_3−*X*_PW_12_O_40_/Fe-SiO_2_ nanocatalyst, (a) precursor and (b) calcined catalyst.

Figure 4 shows SEM pictures of the precursor (a) and calcined catalyst (b). After calcination catalyst particle aggregated together and formed a spherical shape and more uniform particles which are beneficial to the activity and augmenting the surface of the catalyst that exhibited a large amount of aggregates than the precursor. The measured BET surface areas are 237.5 m^2^ cm^3^ g^−1^ for Cs_*X*_H_3−*X*_PW_12_O_40_/Fe-SiO_2_ catalyst and corresponded pore volume is 0.7672 cm^3^ g^−1^ obtained from analysis of the desorption using the BJH (Barrett-Joyner-Halenda) method. The particle size could be calculated by Scherer-equation form XRD pattern (Figure 2). It is clear that the catalyst particle size was in nanodimension (45 nm). The Cs_*X*_H_3−*X*_PW_12_O_40_/Fe-SiO_2_ catalyst was characterized with SEM (Figure 4). It is obvious in this figure that the crystal sizes were from 38–47 nm. This result confirmed the obtained results studied by using the Scherrer equation.

### 4.2. Calculation of Rate Constant, Activation Energy, and Preexponential Factor

**Figure 5:**
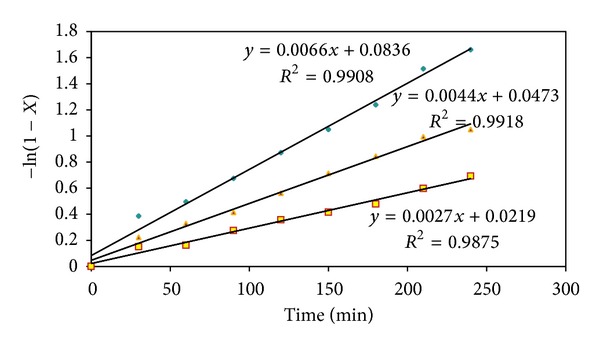
Plots of −ln⁡(1 − *X*) versus time (min) at temperatures 60, 55, and 50°C for reaction of sunflower oil with methanol.

Plots of −ln⁡⁡(1 − *X*_ME_) versus (*T*) are given in Figure 5. Rate constant has been calculated using Figure 5. And activation energy is calculated through the Arrhenius equation:

(4) k=Ae−Ea/RT  

(5) ⇒ln⁡k=ln⁡A−EaRT,

where (*k*) is the reaction constant, *A* is the frequency or preexponential factor, *E*_*a*_ is the activation energy of the reaction, *R* is the gas constant, and *T* is the absolute temperature.

**Figure 6:**
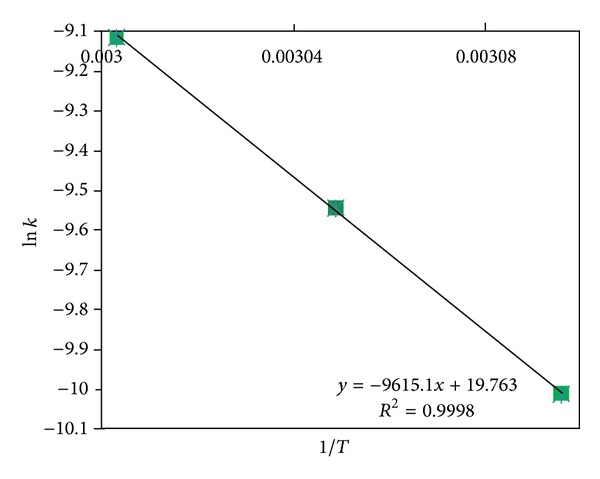
Arrhenius plot of ln⁡*k* versus 1/*T* for reaction of sunflower oil with methanol.

Therefore, plots of ln⁡*k* versus 1/*T* are given in Figure 6. Activation energy (*E*_*a*_) and preexponential factor have been calculated using Figure 6 to be 79.805 kJ/mol and 8.9 × 10^8^ kJ/mol, respectively. Based on the proposed kinetic model, the kinetic parameters for this catalyst were determined. The experiments demonstrate that the reactions follow first-order kinetics. The proposed kinetic model describes the experimental results well and the rate constants follow the Arrhenius equation.

### 4.3. Thermodynamic Parameters (Δ*S* and Δ*H*)

**Figure 7:**
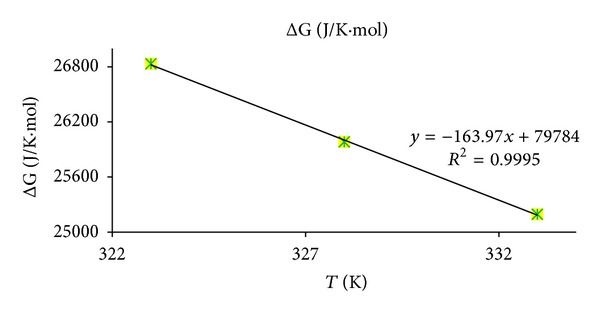
Plot of Δ*G* (J/K·mol) versus *T* (K) for reaction of sunflower oil with methanol.

Based on the definition of Gibbs energy free using (7) and using linear plot of ln⁡*k*_eq_versus ln⁡1/*T* which is given in Figure 7 and by using (7) the respective values of Δ*S* and Δ*H* were calculated which are 0.0197 kJ/mol, 79.784 kJ/Kmol respectively:

(6) $$\begin{array}{c} \Delta G=-RT\ln k_{\text{eq}} \end{array}$$

(7) $$\begin{array}{c} \ln k_{\text{eq}}=\frac{\Delta S}{R}-\frac{\Delta H}{RT}. \end{array}$$

From the results (Table 2) of the thermodynamic parameters it can be found that the transesterification reaction is an endothermic reaction and, with increasing temperature, reaction rate increases. Moreover, enthalpy and entropy change are not affected by methanol concentration due to its excess [26].

## 5. Conclusions

The magnetic Cs_*X*_H_3−*X*_PW_12_O_40_/Fe-SiO_2_ nanocatalyst was prepared for biodiesel production. Experimental conditions were varied as follows: reaction temperature 328–338 K, methanol/oil molar ratio = 12/1, and the reaction time 0–240 min. Thermodynamic properties such as Δ*S* and Δ*H* were successfully determined from equilibrium constants measured at different temperature. Activation energy (*E*_*a*_) and preexponential factor have been calculated to be 79.805 kJ/mol and 8.9 × 10^8^ kJ/mol, respectively. The experiments demonstrate that the reactions follow first-order kinetics. Also notably, recovery of the catalyst can be achieved easily with the help of an external magnet in a very short time (<20 seconds) with no need for expensive ultracentrifugation.

## Figures and Tables

**Table 1:** Concentration of methyl ester (based on mole fraction) at temperatures of 60 (A), 55 (B), and 50°C (C), respectively.

Reaction time (s)		*X*_ME_		−ln (1 − *X*_ME_)		
	A	B	C	A	B	C
0	0.00	0.00	0.00	0.000000	0.000000	0.000000
30	0.32	0.24	0.14	0.385660	0.274437	0.150823
60	0.40	0.28	0.15	0.510826	0.328504	0.162519
90	0.42	0.34	0.24	0.590000	0.415515	0.274437
120	0.5819	0.43	0.30	0.872035	0.562119	0.356675
150	0.63	0.51	0.34	0.994252	0.713350	0.415515
180	0.70	0.57	0.38	1.203973	0.843970	0.478036
210	0.75	0.63	0.45	1.386294	0.994252	0.597837
240	0.81	0.65	0.50	1.660731	1.049822	0.693147

*X*_ME_: concentration of methyl ester.

−ln (1 − *X*_ME_): triglyceride concentration.

**Table 2:** Calculated values of thermodynamic parameters.

*T* (K)	Δ*G* (kJ/K·mol)	Δ*H* (kJ/K·mol)	Δ*S* (KJ/mol)
323	26.83272		
328	25.98118	79.784	0.0197
333	25.19303		
